# A Case Study of the Relationship Between Vegetation Coverage and Urban Heat Island in a Coastal City by Applying Digital Twins

**DOI:** 10.3389/fpls.2022.861768

**Published:** 2022-04-26

**Authors:** Yansu Qi, Han Li, Zonglin Pang, Weijun Gao, Chao Liu

**Affiliations:** ^1^Innovation Institute for Sustainable Maritime Architecture Research and Technology, Qingdao University of Technology, Qingdao, China; ^2^School of Environmental and Municipal Engineering, Qingdao University of Technology, Qingdao, China; ^3^Faculty of Environmental Engineering, The University of Kitakyushu, Kitakyushu, Japan

**Keywords:** fractional vegetation cover, NDVI, urban heat island, forest protection, digital twins, coastal city

## Abstract

While urban vegetation affects the urban thermal environment directly, the effects of different plant layouts and vegetation cover on urban microclimate regulation are different. This study has applied digital technologies to advance urban environmental research and forestry analysis. With a focus on a coastal city located on the eastern coast of the North Temperate Zone as a study area, this study collected the Landsat archive satellite remote sensing image data covering the study area in 2000–2020 and analyzed the temporal and spatial distribution characteristics of vegetation coverage, land surface temperature, and urban heat island (UHI) ratio index. The study results included the following findings: (1) The area of high fractional vegetation cover (FVC) (0.8–1.0) in the study area is increasing. Those areas are located in the mountain forests in the near-coastal area. The lowest temperature was also detected in the mountain area. (2) The distance from the coastline causes a negative correlation between land surface temperature and FVC. The land surface temperature in the regions with a distance of more than 25 km from the coastline decreases obviously with increasing FVC in summer. However, the correlation between the land surface temperature and FVC showed a slight change in the winter period. (3) UHI ratio index decreases along with the area of high FVC (H-FVC) area. The influence of ocean climate on seasons is different, which results in the reduced effect of the H-FVC area and differences in the UHI ratio index. (4) The distance from the coastline should be considered as an important factor in the forestry development planning of the coastal cities.

## Introduction

As an important part of the Earth’s ecosystem, land vegetation is an indispensable natural resource for human survival and development (Jones et al., 2009). Fractional vegetation cover (FVC) reflects the proportion of vegetation in the vertical projected area of the ground per unit area. It is an important standard to balance the change in the ecological environment. Besides, it is also an important basis for national natural resource management and macro-control (Carlson and Ripley, 1997). Urban vegetation directly affects the urban thermal environment. The effects of different vegetation coverage on urban microclimate regulation are different. Many scholars have compared the cooling effects of vegetation in several cities, such as Hong Kong, Madison, and Munich (Taha, 1997; Ng et al., 2012; Alavipanah et al., 2015; Ziter et al., 2019). There are some differences in the cooling effects of different vegetation coverage in cities in different countries. Although the cooling thresholds of vegetation coverage vary in different cities, they all show that when the vegetation coverage is too low, the effect of vegetation on reducing land surface temperature (LST) is weak. Only when a certain amount of vegetation is reached can it be effectively cooled, and a larger vegetation coverage is more conducive to cooling.

Digital twin (DT) technology is a group of virtual information structures, which describe a potential or actual physical manufacturing product from the micro atomic level to macro geometric level. Many industries have adopted DTs to realize many applications, especially in manufacturing (Leng et al., 2021), aerospace industry (Liu et al., 2021), and agriculture (Pylianidis et al., 2021). DT has been extended to the construction field, including the design stage, construction stage, and operation and maintenance stage (Jiang et al., 2021b). DTs of related existing projects, surroundings, and environments can be established to assist decision-making and design, e.g., DT cities (Jiang et al., 2021a; Shahat et al., 2021). By collecting data from the physical world and parsing the data into understandable expressions, DT can assist in complex and sustainable urban planning considering various factors (Jiang et al., 2021a; Pesantez et al., 2021). The traditional methods of vegetation coverage survey mainly rely on field measurement, including the visual estimation method, sampling method, instrument method, model method, and so on. However, the ground survey can only monitor the vegetation coverage in small areas, which is a time-consuming, heavy workload and is limited by many conditions. In recent years, with the continuous development and maturity of remote sensing technology, it is used to study large-scale areas. Remote sensing data are used in many studies to detect changes in vegetation coverage at different spatial scales and discuss their correlation with climate factors (Xin et al., 2008; Vijith and Dodge-Wan, 2020; Gu and Wei, 2021). Computer science has been applied in various disciplines, including artificial intelligence, digital twinning, and deep learning (Fang and Liang, 2003; Zhang et al., 2020; Liu et al., 2022). With the development of science and technology, the traditional field quadrat survey method has gradually changed to the use of digital technology (Ding et al., 2021; Guo et al., 2021). Remote sensing and computer technologies are used to identify and analyze vegetation cover (Mirsanjari et al., 2021; Sun et al., 2021).

Urban characteristics change along with a process of urbanization, which may affect the role of vegetation in changing the urban thermal environment. Increasing urban vegetation is an important means to alleviate the urban heat island (UHI) effect (Akbari and Rose, 2008). However, systematic studies on the relationship between vegetation change and urban thermal environment in the long-term urbanization process are rarely reported. In terms of the spatial pattern of urban thermal environment, Min et al. (2019) found that areas with high land surface temperature are mainly distributed in areas with high construction degree and dense population, while areas with high vegetation or water cover have a lower surface temperature. In recent decades, more and more attention has been paid to the role of surface temperature for UHIs (Voogt and Oke, 2003).

This study uses DT technology to investigate the effect of vegetation coverage on the urban thermal environment and UHI for the coastal city. An archive collection of satellite remote sensing images covering the study area of Qingdao from 2000 to 2020 were used as the data source. Pixel decomposition model (PDM) based on normalized difference vegetation index (NDVI) was used to analyze changes in vegetation coverage. Similar to the neural network applications, our approach was determined to use a method based on a radiance transfer equation, which is aimed to retrieve land surface temperature. Based on the vegetation information extracted from the PDM model and the land surface temperature, the effect of vegetation change on the land surface temperature and UHI radio index were analyzed both spatially and temporally.

The major contributions of this study are expressed as follows: (1) This study evaluates vegetation coverage, land surface temperature, and UHI ratio index in summer and winter for the past 20 years and provides the results of coastal area in Qingdao temporally and spatially. (2) It offers a method of classifying areas using distance from the coastline to research the effect of vegetation change on land surface temperature and the UHI effect in coastal cities. (3) It provides a useful case study for coastal city research by using DT technology for data collection, data visualization, and data analysis to make decision in urban planning. (4) Based on the analysis and discussion of the results from different regions, this study proposed the accessible planning strategies to the Territorial Spatial Planning of Qingdao city (2021–2035).

## Data and Methods

**FIGURE 1:**
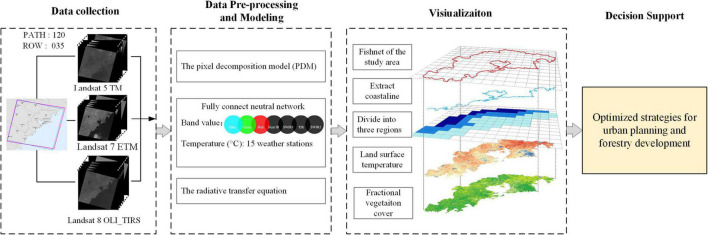
Research procedure of applying Digital Twins.

Digital twin technology is the comprehensive use of information technologies, such as perception, calculation, and modeling, to describe, diagnose, predict, and make decisions in physical space through software definition and to realize the interactive mapping between physical space and cyberspace. This study investigates the effect of vegetation coverage on the urban thermal environment and UHI for the coastal city by following several processes. Figure 1 demonstrates the four processes that include data collection from real space and creating a dataset, calculation and visualization of results based on the past data, further analysis of results and data for decision support, and optimization strategies. At first, Landsat images are used as a data set to collect band information from the images. Then, the vegetation coverage information, land surface temperature, and UHI ratio index (URI) are calculated by the remote sensing technologies. The data are visualized in 2D mappings and analyzed to learn about their differences. Finally, the results and data are further analyzed to serve the decision support step and suggest corresponding optimization strategies.

### Study Area and Data Collection

**FIGURE 2:**
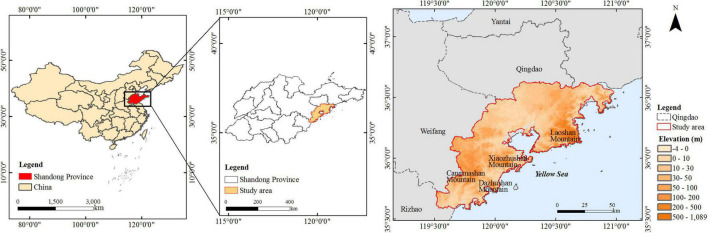
Location and elevation data of study area.

Qingdao is located on the southeast coast of the Shandong Peninsula. It has a complex terrain, affected by typical temperate monsoon climate and rich vegetation types. As shown in Figure 2, Qingdao is a coastal hilly city with high terrain in the east and low terrain in the west, uplifted in the north and south sides, and low depression in the middle area. It is located in the warm temperate zone of the Northern Hemisphere. Affected by Jiaozhou Bay and the Yellow Sea, Qingdao also has the characteristics of a marine climate. Moreover, Qingdao is a city with an inner bay, developing around Jiaozhou Bay; the ecological environment around Jiaozhou Bay has also been paid attention to more research (Qin and Zhang, 2021). According to the “Territorial Spatial Planning of Qingdao city (2021–2035),” the urban area around Jiaozhou Bay is a core of the city development. The study area includes the urban areas around Jiaozhou Bay, including Shinan, Shibei, Laoshan, Licang, Chengyang, Jimo, the West Coast New Area, and Jiaozhou. The area is about 6,320 km^2^. In the past two decades, Qingdao has experienced profound economic development, with significant urbanization effects. On the one hand, the impact of vegetation coverage change on the heat island effect in Qingdao in recent years can provide a reference for a new round of urban sustainable planning. On the other hand, it is also of great significance to explore the response process of vegetation to climate change in the coastal city of the eastern monsoon region of China.

The archival data of the study area were collected and analyzed through digital methods to provide optimization strategies for the new round of urban planning. Therefore, we collected Landsat satellite images, elevation data, and meteorological data from the real world as data sets. Vegetation coverage and land surface temperature data were assessed by remote sensing images of three Landsat satellites (Landsat 5 TM, Landsat 7 ETM, and Landsat 8 OLI) from 2000 to 2020, as shown in Figure 2. The image path and row of the Landsat images were 120 and 35, respectively. This study obtains two images with different dates within each year. The dates and some technical characteristics for the satellite data were acquired from the USGS Earth Explorer. We used 15 temperature observation data from meteorological stations that are covered by remote sensing images that are used to extract temperature to build the data set. The remotely sensed images were acquired at different times, so the temperature data used are also the corresponding satellite transit time temperatures. Remote sensing data are greatly influenced by clouds and weather (Gallo et al., 2011). Considering that the impacts of the UHI phenomenon on people’s daily life are most apparent in summer (June to August) and winter (December to February), cloud-free satellite images were selected to calculate the average LST (Wu et al., 2022). If cloud-free satellite images were not available for the classification and the LST calculation in 2000–2020, the images of adjacent years with low cloud coverage (lower than 5%) were selected (Simwanda et al., 2019). The temperature data for the same period were obtained from the weather station. Temperature data of 15 meteorological stations within the range of remote sensing images from 2000 to 2020 were used as real values to construct and test the remote sensing estimation model of temperature corresponding to Landsat satellite transit time (UTC 02:30).

### Pixel Decomposition Model

Compared to the traditional methods, such as field survey, quadrat survey, and other adopted means, there are certain limitations for the large areas. FVC is calculated by *PDM* and divided FVC into different classifications through collecting the NDVI, which is the most popular vegetation index. Its formula is as follows:


(1) $$N\,D\,V\,I=\left(p_{n\,i\,r}-p_{r}\right)/\left(p_{n\,i\,r}+p_{r}\right)$$

where *p*_*nir*_ is the reflection value of the near-infrared band, *p*_*r*_ is the reflection value of the red band, *NDVI*_*min*_ is the NDVI value of pure bare soil, and *NDVI*_*max*_ is the NDVI value of high vegetation coverage.

The PDM method of pixel (Leprieur et al., 1983) consists of dividing the ground object composition of the pixel into vegetation and non-vegetation and decomposing the mixed pixel by obtaining spectral information from pure and non-pure vegetation pixel (Zhang et al., 2019). The NDVI pixels can reflect growth conditions and cover changes of vegetation, and it has widely been calculated using Equation (4) to invert FVC (Gutman and Ignatov, 1998).


(2) $$F\,V\,C=\frac{\left(N\,D\,V\,I-N\,D\,V\,I_{s\,o\,i\,l}\right)}{\left(N\,D\,V\,I_{v\,e\,g}-N\,D\,V\,I_{s\,o\,i\,l}\right)}$$

where NDVI refers to the NDVI value of mixed pixels that need to calculate vegetation coverage,*NDVI*_*soil*_ is the vegetation index of the bare soil pixels, and *NDVI*_*veg*_ is the vegetation index of whole vegetation cover pixels.


(3) $$N\,D\,V\,I_{s\,o\,i\,l}=\frac{\left(F\,V\,C_{\max}\,\text{×}\,N\,D\,V\,I_{\min}-F\,V\,C_{\max}\,\text{×}\,N\,D\,V\,I_{\max}\right)}{\left(F\,V\,C_{\max}-F\,V\,C_{\min}\right)}$$

(4) $$N\,D\,V\,I_{v\,e\,g}=\frac{\left(1-F\,V\,C_{\min}\right)\,\text{×}\,N\,D\,V\,I_{\max}-\left(1-F\,V\,C_{\max}\right)\,\text{×}\,N\,D\,V\,I_{\min}}{\left(F\,V\,C_{\max}-F\,V\,C_{\min}\right)}$$

where *NDVI*_*veg*_ is a pure vegetation-covered pixel value, which will change at different spatial-temporal scales due to the influence of vegetation types. *NDVI*_*veg*_ of different land-use types can be calculated based on the land use map. *NDVI*_*soil*_ is the NDVI value of pure soil pixels. The theoretical value *NDVI*_*soil*_ should be close to zero, but *NDVI*_*soil*_ will change with time and space due to atmospheric influence and different conditions, such as surface temperature, humidity, roughness, and soil type. Therefore, it is not advisable to adopt a fixed *NDVI*_*soil*_ value. By analyzing the histogram distribution of NDVI extracted from Landsat data, *NDVI*_*soil*_ and *NDVI*_*veg*_ of a certain frequency were selected from the NDVI frequency accumulation table.

The selection of *NDVI*_*veg*_ and *NDVI*_*soil*_ greatly affects the inversion precision of FVC (Wu and Murray, 2003). In this study, 5 and 95% confidence intervals of 0.06 and 0.6 were selected as *NDVI*_*soil*_ and *NDVI*_*veg*_ values, respectively. NDVI and FVC can reflect the growth and distribution of vegetation in the area. To analyze the temporal and spatial changes of vegetation coverage better, the vegetation coverage of the study area is classified into 5 grades, where 0-0.2 is bare land and water (BL and W), 0.2-0.4 is low FVC (L-FVC), 0.4-0.6 is a secondary low FVC (SL-FVC), 0.6-0.8 is a medium FVC (M-FVC), and 0.8–1 is a high FVC (H-FVC).

### Methodology of Land Surface Temperature Inversion

The radiation information received by the satellite sensor includes the reflected radiation information of the ground object after atmospheric attenuation and the atmospheric range radiation information. Therefore, it is necessary to restore the real reflectance of the target object through an atmospheric correction to eliminate the influence of the atmosphere on the radiance of the ground object. Radiometric calibration aims to convert the dimensionless digital number (DN) value recorded by the sensor into the relative value of apparent radiance or apparent reflectance of the corresponding object with practical physical significance. For Landsat TM/ETM+/OLI and TRIS data, the DN value is converted into a radiance image according to the absolute calibration coefficients. Since the airborne scanning line corrector carried by ETM+, carried by Landsat7, failed in May 2003, all the acquired images were seriously lost. Given this situation, this study used the plug-in of ENVI 5.3 software to repair the data. Thermal band data can also be converted from spectral radiance to effective at-satellite temperature. The fundamental principle of this algorithm is based on the thermal infrared radiative transfer equation, which removes the influence of the atmosphere on the thermal radiation during the radiative transfer process and thus obtains the land surface temperature more accurately. The algorithm is widely available and can be used for thermal infrared remote sensing data on any sensor. The thermal infrared radiative transfer is calculated according to the following equation (Schneider and Mauser, 1996):


(5) $$L_{\mathrm{sen}}=\left(\varepsilon \,\text{B}\,\left(T_{s}\right)+\left(1-\varepsilon \right)\,L_{d}\right)\,\tau +L_{u}$$

where, *L*_sen_ is the satellite measured thermal radiance (W⋅m^–2^⋅sr^–1^⋅μ m^–1^), ε is the emissivity of the land surface, *B*(*T*_*s*_) is the radiative brightness of the blackbody in the thermal infrared band of the *T*_*s*_, *B* is the Plank function, *T*_*s*_ is the land surface temperature (K), *L*_*u*_is the upward longwave atmospheric radiation, *L*_*d*_is the downward longwave atmospheric radiation, and τ is the atmospheric transmissivity. A conversion formula of LST can be calculated according to the following formula (Mustard et al., 1999; Chander and Markham, 2003):


(6) $$T=\frac{K_{2}}{I\,n\,\left(\left(\frac{K_{1}}{B\,\left(T_{s}\right)}\right)+1\right)}$$

where *K*_*2*_ and *K*_*1*_ are the calibration constant 2 and constant 1 in kelvin of Landsat 5/7/8, respectively.

As an important part of artificial intelligence, the artificial neural network has widely been applied in the fields of pattern recognition, function approximation, remote sensing classification of land use, and remote sensing inversion of biophysical parameters (Hagan and Menhaj, 1994). Till now, a variety of neural network models have been established, among which the most widely used is the error backpropagation neural network, referred to as a fully connected neural network. The collected remote sensing images and temperatures from 15 weather stations for the past 20 years are used as the data set, and a fully connected neural network is built to train the data. The fully connected neural network with three hidden layers was used in the study. The sample data sets of multispectral and thermal infrared reflectance in Landsat TM/ETM+/OLI images of 15 weather stations were taken as the input of the network (band 1–7 of Landsat 5; band 1–7 of Landsat 7; and band 2–7 and band 10 of Landsat 8). The land surface temperature data of 15 weather stations were taken as the output of the network.

**FIGURE 3:**
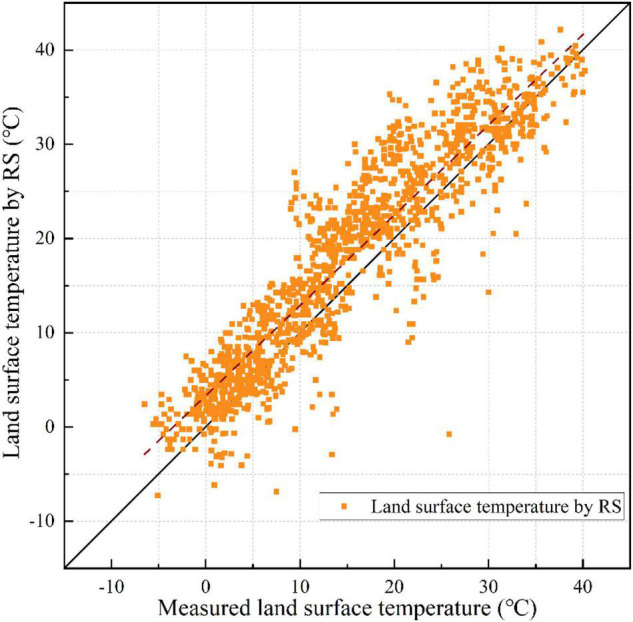
Correlation between the measured LST (*x*-axis) and LST by RS (*y*-axis) in radiative transfer equation method.

Mean absolute error (MAE) represents the mean of the absolute error between the predicted and observed values. Root mean square error (RMSE) measures the deviation between the predicted and true values and is more sensitive to outliers in the data. To determine the accuracy of the method of inversion LST, the same time hourly value of 15 meteorological stations from 2000 to 2020, which are covered in the remote sensing image, was extracted and compared with the inversion LST. After the operation based on a fully connected neural network, MAE was 4.67 and RMSE was 3.89. To compare the results with those of the neural network training, MAE was also calculated for the inverse performance of the LST of the radiative transfer equation at 15 corresponding weather stations. As shown in Figure 3, MAE was 4.18 and RMSE was 5.54. The neural network method is more influenced by the training data and does not have a particularly strong advantage over the radiative transfer equation method. Therefore, this study uses traditional methods for LST inversion.

### Urban Heat Island

The LST data were first normalized to better compare the differences in UHI intensity in different periods. Afterward, those normalized data were classified into different temperature segmentations. The mean-SD method is an ideal method for temperature classification. SD reflects the deviation value for the average temperature. Urban land surface temperatures were classified into the five temperature segmentation zones (low temperature, secondary low temperature, medium temperature, secondary high temperature, and high temperature). The basic principle of using the mean-SD method for temperature classification is shown in Table 1, where μ is the average temperature and *std* represents the standard deviation of land surface temperature.

**TABLE 1:** Urban heat island temperature classification using mean-standard deviation method.

Land surface temperature classification	Interval of temperature classification
High-temperature zone (HTZ)	*LST*_*i*_ > μ + *std*
Secondary high-temperature zone (SHTZ)	μ + 0.5*std* < *LST*_*i*_ ≤ μ + *std*
Medium temperature zone (MTZ)	μ − 0.5*std* < *LST*_*i*_ ≤ μ + 0.5*std*
Secondary low-temperature zone (SLTZ)	μ − *std* < *LST*_*i*_ ≤ μ − 0.5*std*
Low-temperature zone (LTZ)	*LST*_*i*_ < μ − *std*

The URI is used to depict the development degree of heat island (Xu et al., 2011). The greater the URI is, the more severe the heat island phenomenon is.


(7) $$U\,R\,I=\frac{1}{100\,m}\,\sum_{i=1}^{m}\omega_{i}\cdot p_{i}$$

where *m* is the number of LST grade (*m* = 5), ω is weighted value, the ω values of HTZ, SHZT, MTZ, SLTZ, and LTZ are 5, 4, 3, 2, and 1, respectively, and *p* is area percentage.

## Results and Analysis

In this section, the FVC and LST from 2000 to 2020 are calculated according to the “Pixel Decomposition Model” and “Methodology of Land Surface Temperature Inversion” sections, respectively. The data are visualized in 2D maps and analyzed to learn about their differences. FVC and LST data were represented in virtual space to support the decision supporting DT. Then, the areas and proportion of the LST classifications were calculated. The LST was classified according to the classification method in the “Urban Heat Island” section. Then, the proportion of classification zones and URI was calculated at the same time, respectively.

### Distribution of Fractional Vegetation Cover

By using the established PDM, the vegetation coverage area is calculated and visualized. The FVC in the study area is expressed by NDVI that was extracted from remote sensing data. As a normal vegetation growth cycle, vegetation growth begins in March and April, reaches a peak of the NDVI in the summer months of June and July, and declines in the winter months of December and January (Chu et al., 2007). Therefore, two remote sensing images are selected per year, one in June or July as the highest growing season and the other in January or December as the lowest growing season. The FVC is classified into five classes: (1) the FVC distributed in 0-0.2 represented the BL and W, (2) the FVC distributed in 0.2-0.4 represented the L-FVC, (3) the FVC distributed in 0.4-0.6 represented the SL-FVC, (4) the FVC distributed in 0.6-0.8 represented the M-FVC, and (5) the FVC distributed in 0.8-1.0 represented the H-FVC.

**FIGURE 4:**
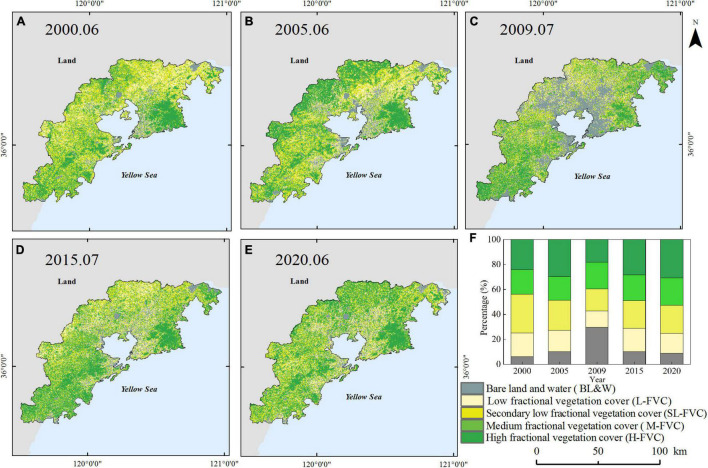
Classification of fractional vegetation cover in summer from 2000 to 2020 **(A)** 2000.06, **(B)** 2005.06, **(C)** 2009.07, **(D)** 2015.07, **(E)** 2020.06, and **(F)** the percentage of different classification from 2000 to 2020.

**FIGURE 5:**
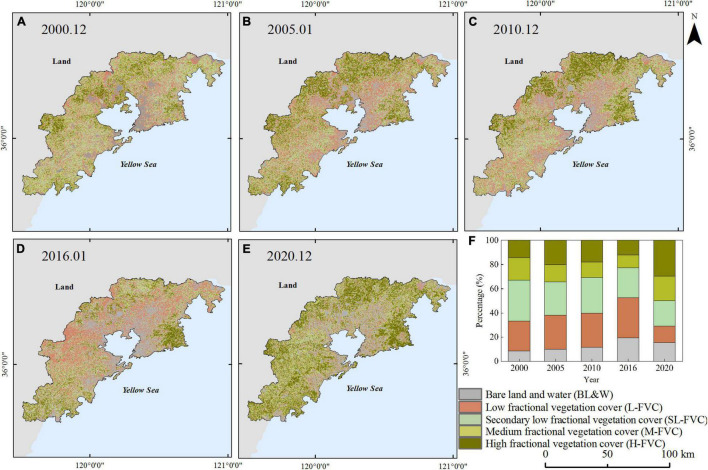
Classification of fractional vegetation cover in winter from 2000 to 2020 **(A)** 2000.12, **(B)** 2005.01, **(C)** 2010.12, **(D)** 2016.01, **(E)** 2020.12, and **(F)** the percentage of different classification from 2000 to 2020.

The spatial distribution of FVC is shown in Figures 4, 5. The study area is a coastal city, and the urban construction mainly extends along Jiaozhou Bay area to the east and west coastal areas. In general, vegetation strongly depends on the changes in climate setting in the area and land use. The H-FVC and M-FVC are distributed in the peripheral areas of the study area. The green pixels represent the H-FVC and M-FVC. According to Figure 2, Laoshan, Xiaozhushan, Dazhushan, and Cangmashan mountains are distributed in the study area, which is mainly covered by forests. As shown in Figures 4, 5, the mountain regions are covered by pixels that represent H-FVC and M-FVC areas. The H-FVC area is mainly covered by mountainous forest lands, while the dominating land cover type of the M-FVC area is agricultural land. The H-FVC proportion varies in the range of 24.1–30.85% and the range of 12.1–29.52%. It can be shown that the area of H-FVC in summer is higher than in winter. The FVC of evergreen trees does not change with the season, and the FVC of deciduous trees and crops decreases in winter, thus the overall proportion of H-FVC is smaller than the proportion of summer. From 2000 to 2020, the H-FVC proportion shows an upward trend during the winter and summer. According to the data of Qingdao weather station (Station ID: 54857), the recent 20 years annual mean temperature increased from 13.22 to 14.15°C. Under the condition of constant precipitation content, the change of temperature can affect the growth of the plant. The increase of temperature can prolong the growth time of plants and promote the growth and development of plants, which is reflected in the increase of vegetation coverage.

### Distribution of Land Surface Temperature

**FIGURE 6:**
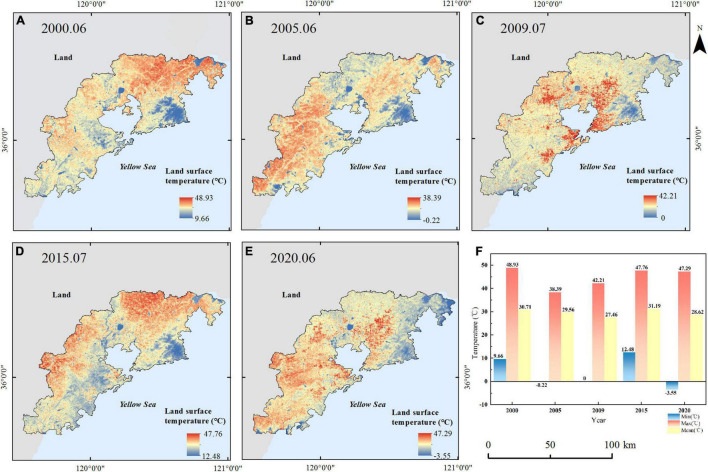
Land surface temperature (LST) in summer from 2000 to 2020 **(A)** 2000.06, **(B)** 2005.06, **(C)** 2009.07, **(D)** 2015.07, **(E)** 2020.06, and **(F)** the max, mean, and min LST in summer from 2000 to 2020.

**FIGURE 7:**
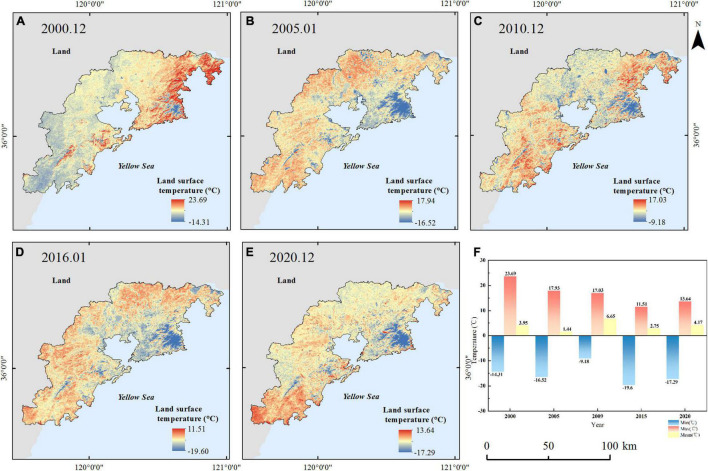
Land surface temperature (LST) in winter from 2000 to 2020 **(A)** 2000.12, **(B)** 2005.01, **(C)** 2010.12, **(D)** 2016.01, **(E)** 2020.12, and **(F)** the max, mean, and min LST in winter from 2000 to 2020.

The LST distribution is obtained from the Landsat data by the traditional retrieving method based on the radiance transfer equation. Location, landscape, and distance from the ocean affect the urban thermal environment (Zheng et al., 2014; Estoque et al., 2017). As shown in Figures 6, 7, the altitude of the eastern and western regions in the study area is higher than those in the central region, which makes an obvious difference in the distribution of the regional thermal environment. It can be seen that the lowest temperature of the study area is distributed in the mountain area with a higher FVC. The relatively low values of LST are distributed in the forest land areas of Laoshan, Dazhushan, Xiaozhushan, and Cangmashan mountains in the study area. Vegetation changes can directly affect regional land surface temperature; the cooling effect is affected by geographical conditions (Li et al., 2012). The decline range of land surface temperature can be noted along with a varying increase in vegetation coverage. In summer, the average temperatures and the temperature difference vary in ranges of 27.79–31.19°C and 35.26–50.84°C, respectively (Figure 6F). In winter, the average temperatures and the temperature difference vary in ranges of 1.44–6.65°C and 26.21–28°C, respectively (Figure 7F).

### Urban Heat Island Ratio Index

**FIGURE 8:**
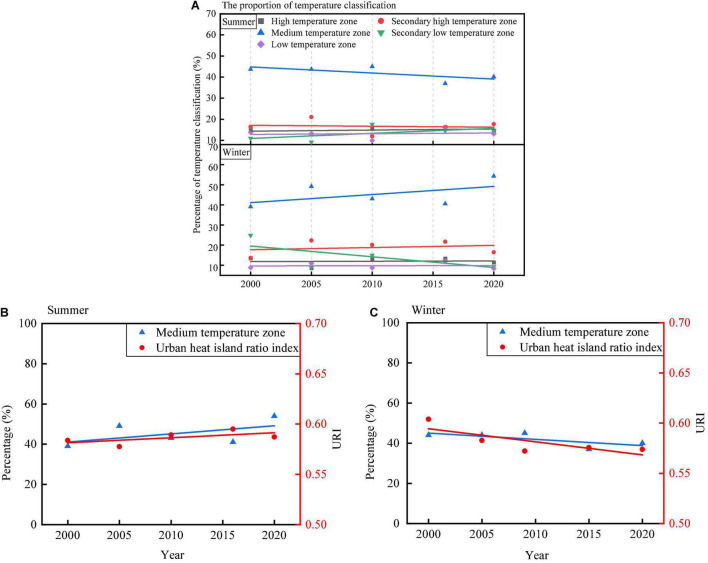
Temporal change of urban heat island ratio index **(A)** the proportion change of different classificaiton in winter and summer from 2000to 2020, **(B)** the medium temperature proportion and URI in summer from 2000to 2020, and **(C)** the medium temperature proportion and URI in winter from 2000to 2020.

According to Table 1 and the mean-SD method, the LST of the study area is classified into five classifications: (1) high temperature zone (HTZ), (2) secondary high temperature zone (SHTZ), (3) medium temperature zone (MTZ), (4) secondary low temperature zone (SLTZ), and (5) low temperature zone (LTZ). As shown in Table 2, the MTZ proportion is in the range of 36.93–45% and 39.09–54.35% in summer and winter, respectively. The proportion of MTZ is the largest in the study area. As shown in Figure 8A, the MTZ proportion shows different trends in summer and winter. From 2000 to 2020, the MTZ proportion shows a decreasing trend in summer and an increasing trend in winter. The SLTZ proportion shows an increasing trend in summer and a decreasing trend in winter. The three remaining temperature classifications (HTZ, SHTZ, and LTZ) showed no obvious trend in winter and summer. URI is calculated by the LST classified into five classifications (HTZ, SHTZ, MTZ, SLTZ, and LTZ) according to Table 1. The calculated URI based on five classifications of summer and winter is listed in Table 2. The URI varies in the range of 0.577–0.595 and 0.572–0.604 in summer and winter, respectively. The URI shows different trends in summer and winter. From 2000 to 2020, as shown in Figure 8B, the URI shows a slight increasing trend in summer. As shown in Figure 8C, the URI shows a slight decreasing trend in winter. Coastal cities are influenced by the ocean climate in summer, which also influences the UHI effect.

**TABLE 2:** Area of fractional vegetation cover and land surface temperature segmentation.

Year		Area of FVC segmentation (km^2^)					Area of LST segmentation (km^2^)				
		BL andW	L-FVC	SL-FVC	M-FVC	H-FVC	HTZ	SHTZ	MTZ	SLTZ	LTZ
Summer	2000.06	366.27	1130.73	1872.23	1178.33	1443.78	906.36	982.81	2619.26	657.43	825.51
	2005.06	611.89	1042.845	1462.21	1166.90	1802.48	785.27	1280.25	2657.49	561.73	801.60
	2009.07	1823.94	810.22	1090.41	1320.57	1116.84	952.79	737.38	2773.14	1087.06	611.91
	2015.07	617.73	1159.76	1379.00	1279.08	1750.11	1008.44	1008.75	2284.25	898.59	985.69
	2020.06	540.87	998.00	1394.44	1344.36	1908.04	907.56	1093.14	2479.23	899.82	805.97
Winter	2000.12	508.29	1479.30	2026.24	1114.43	863.08	809.12	814.85	2338.86	1490.14	530.20
	2005.01	603.30	1717.48	1674.91	862.25	1228.40	522.25	1359.04	2993.18	542.83	669.12
	2010.12	722.36	1732.53	1807.00	800.94	1099.43	808.89	1238.82	2650.40	925.88	538.31
	2016.01	1197.54	2057.59	1534.55	652.84	743.18	824.99	1338.22	2504.20	764.66	735.65
	2020.12	948.63	844.41	1307.38	1259.15	1826.06	705.17	1017.44	3361.66	581.63	519.81

*FVC, fractional vegetation cover; LST, land surface temperature; BL and W, bare land and water; L-FVC, low fractional vegetation cover; SL-FVC, secondary low fractional vegetation cover; M-FVC, medium fractional vegetation cover; H-FVC, high fractional vegetation cover; HTZ, high temperature zone; SHTZ, secondary high temperature zone; MTZ, medium temperature zone; SLTZ, secondary low temperature zone; LTZ, low temperature zone.*

## Discussion

Analyzing the results for decision support is the last and most important part of DT. In this section, results are presented for processes and analysis, and strategies are proposed for decisions based on the results. According to the “Results and Analysis” section, changes in vegetation cover and thermal environment in the study area show different trends in summer and winter. The effects of vegetation change on land surface temperature and UHI radio index are analyzed spatially and temporally for the past 20 years in different seasons.

### Spatial Analysis Between Vegetation Cover and Land Surface Temperature

**FIGURE 9:**
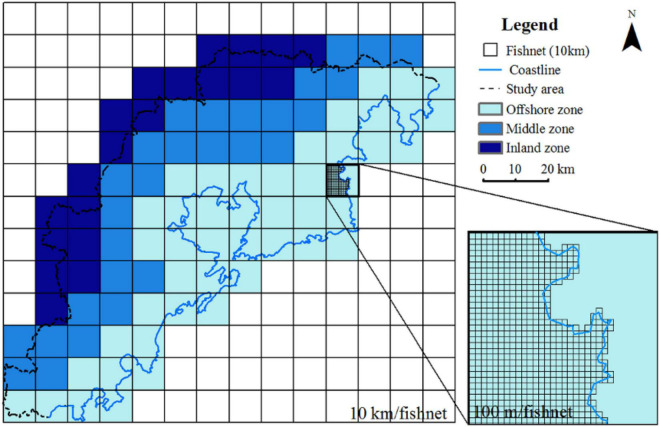
Division of the study area according to the distance from the coastline.

Vegetation reduces the solar radiation incident on the ground by absorbing solar radiation from the surface and reduces evaporation by increasing the humidity of the surrounding air through canopy shading, thus leading to a decrease in the environment temperature. Therefore, changes in vegetation can directly affect the regional land surface temperature. For the study area located in the coastal region of the Yellow Sea, the ocean has a direct impact on the urban thermal environment of the coastal city. The urban thermal environment of the coastal area is affected by sea and land winds. Considering the influence of this factor, the study area is divided into three zones according to the distance to the coastline, as shown in Figure 9. The three zones include the offshore zone (≤10 km), middle zone (between 10 and 25 km), and inland zone (more than 25 km). The study area was divided by fishnets with a length of 100 m through ArcGIS 10.6. Then, the mean FVC and mean LST corresponding to each fishnet are extracted and statistically analyzed.

**FIGURE 10:**
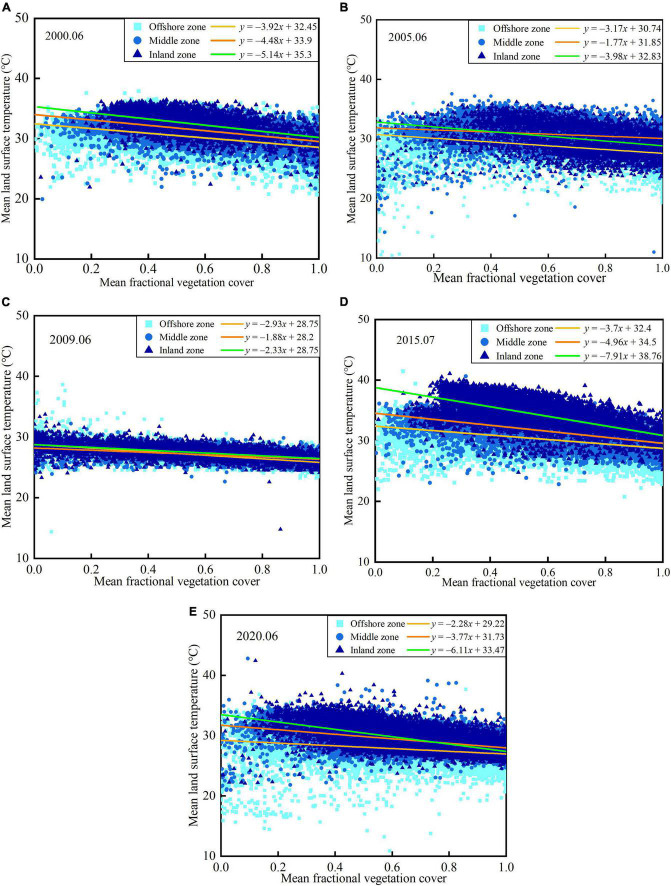
Variation of land surface temperature and fractional vegetation cover in different zones **(A)** 2000.06, **(B)** 2005.06, **(C)** 2009.06, **(D)** 2015.07, and **(E)** 2020.06.

Figure 10 shown the fitting results of summer from 2000 to 2020. The fitting results indicate that there is a negative correlation between mean LST and mean FVC in summer, which illustrates that vegetation has an obvious cooling effect on the land surface. From 2000 to 2020, fitting slopes of the offshore zone and middle zone vary in the range of −3.92 to −2.28 and in the range of −4.96 to −1.77, respectively. Those inland zones vary in the range of −7.91 to −2.33. These results indicate that the negative correlation is more obvious in the inland zone, which results from the effects of ocean climate and sea-land wind on the LST decrease with increasing distance from the coastline. In winter, from 2000 to 2020, the fitting slopes of offshore zone, middle zone, and inland zone vary in the range of −0.24 to 1.23, −4.94 to 0.41, and 0.05 to 3.61, respectively. These results indicate that the distance from the coastline has a slight influence on the correlation between mean LST and mean FVC, which is different from the results in summer. This can be explained by the fact that the influence of sea and land winds is weakened by the inland-oriented northerly wind.

### Temporal Analysis Between Vegetation Cover and Urban Heat Island Effect

When concerning sustainable urban development, forests need to be protected as they are important natural resources for sustaining ecological balance due to the high vegetation cover. The forest has a high vegetation cover. In the study area, the H-FVC area is concentrated in the forest land of Laoshan, Xiaozhushan, Dazhushan, and Cangmashan mountains. The typical vegetation types of the mountains are deciduous oak forest dominated by *Quercus acutissima* and *Quercus variabilis*, as well as temperate coniferous forest dominated by *Pinus densiflora* and *Pinus thunbergii*. H-FVC proportion changes with urban expansion and development. Therefore, the impact of H-FVC proportion on the UHI effect is discussed temporally.

**FIGURE 11:**
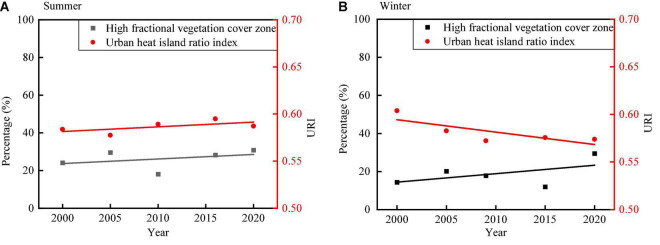
Variations of URI and H-FVC proportion from 2000 to 2020 **(A)** variations of URI and H-FVC proportion in summer and **(B)** variations of URI and H-FVC proportion in winter.

The trends of H-FVC proportion and URI in summer and winter are shown in Figure 11. From 2000 to 2020, the H-FVC proportion shows upward trends in both summer and winter. The URI shows different trends in summer and winter. The URI shows an increasing trend in summer and a decreasing trend in winter. In summer, both H-FVC proportion and UHI show a similar trend. In winter, H-FVC proportion and UHI show an opposite trend. Coastal cities are influenced by the ocean climate in summer, which also influences the UHI effect. The H-FVC area is mainly distributed in the offshore zone significantly influenced by the ocean climate in summer (discussed in the “Spatial Analysis Between Vegetation Cover and Land Surface Temperature” section), which affects the URI, thus leading to different URI trends in summer and winter. In summer, the H-FVC area located in the near-coastal area is relatively weak in regulating the heat island effect because of the influence of ocean climate and sea and land winds. In winter, with the increase of the area of H-FVC, the UHI effect shows a decreasing trend.

### Urban Development Strategy for the Coastal City

In China, a new round of urban planning adjustment is carried out every 5 years, and the study area is currently under urban planning adjustment. As the study area is a metropolitan area under planning, changes in vegetation in the area will cause changes in the thermal environment, which in turn will affect the heat island. Therefore, according to the above analysis, vegetation has an obvious cooling effect, and the region of mountain forest land in H-FVC and coniferous forest should be protected. In the urban development area, the vegetation coverage of the M-FVC and SL-FVC areas should be further improved under the premise of maintaining the current green land and greening rate. Vegetation has a significant cooling effect on the land surface. The negative correlation is more obvious in the inland zone, which results from the effects of ocean climate and sea-land wind on the LST decrease with increasing distance from the coastline. The distance from the coastline has a slight influence on the correlation between the LST and FVC, which, however, is different in summer. This can be explained by the fact that the influence of sea and land winds is weakened by the inland-oriented northerly wind. In summer, the H-FVC area located in the near-coastal area is relatively weak in regulating the heat island effect because of the influence of ocean climate and sea and land winds. In winter, with the increase in the area of H-FVC, the UHI effect shows a decreasing trend. Suggestions are summarized based on the research results as follows.

During the summer period, the vegetation in the inland zone with a distance of ≥25 km from the coastline has an obvious cooling effect on the land surface temperature due to the influence of the marine climate and sea and land winds. From the perspective of forestry development, forest resources should be increased in the inland zone to promote the development of urban greening because forests are important natural resources due to the high vegetation coverage, which well sustains ecological balance. In the study area, the H-FVC area is concentrated in the forest land of the Laoshan, Xiaozhushan, Dazhushan, and Cangmashan mountains. The H-FVC area has a reduced effect on the UHI effect in winter. The protection of forests in the above-mentioned areas should be further strengthened by optimizing the structure of forest species and tree species and improving the quality of standing forests.

Urban forestry is an important element in urban development, and urban forestry planning should be included in the overall planning and design. The study area is a major planning area in the Territorial Spatial Planning of Qingdao city (2021–2035). In the new round of urban and rural planning, green space and forest areas can be optimized according to the phenomenon that the cooling effect of vegetation decreases as the distance from the coastline becomes smaller.

(1) The forestry planning department of Qingdao should determine the trend and scale of urban forestry development according to different geographical and socio-economic conditions. It enables urban forestry and urban planning to develop harmoniously and regulate the urban ecosystem.
(2) In the built-up areas that are located near the sea, the cooling effect of vegetation is influenced by the marine climate, and the green space planning focuses on the microclimate regulation scale at the neighborhood scale.
(3) The cooling effect of vegetation is more significant in the areas that are far from the coastline. Regional green space planning focuses more on the landscape patch scale and should pay attention to the distribution pattern of green space patch size in space.

## Conclusion

Digital twin technology was used in this research to study the effect of vegetation coverage on the urban thermal environment and UHI for the coastal cities. Historical satellite remote sensing images of Qingdao from 2000 to 2020 were used for the base data. The PDM based on the NDVI was used to analyze changes in the vegetation coverage. Compared with the neural network applications, our method is based on the radiance transfer equation, which was determined to retrieve land surface temperature. The effects of vegetation change on surface temperature and the UHI radio index were analyzed both spatially and temporally. With a case study on Qingdao city in China, the distribution of vegetation cover and land surface temperature is analyzed based on the DT technology. Based on the remote sensing data, the correlation between FVC and land surface temperature was analyzed and demonstrated on a series of maps. The following conclusions are obtained:

(1) The area of H-FVC (>0.8) is increasing in the study area. It is mostly concentrated in the forest land of Laoshan, Xiaozhushan, Dazhushan, and Cangmashan mountains located in the near-coastal area. The lowest temperature of the study area was also detected in the mountainous forest areas.
(2) The distance from the coastline influences is the major reason for a negative correlation between the land surface temperature and FVC. The ocean climate and the sea-land wind influence the land surface temperature of different areas of the coastal city. The land surface temperature in the regions with a distance exceeding 25 km from the coastline decreases obviously with increasing fractional vegetation in summer. But the correlation between land surface temperature and FVC showed a slight change in winter. These results can be explained by the influence of ocean climate, which is different between the summer and winter periods.
(3) Urban heat island ratio index decreases with the increase in areas with high fraction vegetation cover(0.8–1.0). H-FVC cover areas are located in the forests of Laoshan, Xiaozhushan, Dazhushan, and Cangmashan mountains, which are mostly situated near the coasts. The influence of the ocean climate on seasons is different, which leads to the reduced effect of the H-FVC area on URI, which varies in different seasons.
(4) The distance from the coastline should be considered as an important environmental factor in the forestry development planning of coastal cities. H-FVC of the forest has an obvious cooling effect. The regions of coniferous forests situated in Laoshan, Xiaozhushan, Dazhushan, and Cangmashan mountains should be protected.

Therefore, to ensure sustainable development in Qingdao, it is necessary to adhere to the environmental policies, including land reclamation to forests and grass type. Besides land-use type policies, it is recommended to strengthen the treatment of enterprises to avoid environmental pollution and to control the expansion of the urban land area, as well as population growth. Finally, the increase in publicity regarding the environmental protection policies, raising public awareness concerning ecological protection, would support the balance between nature and humans.

## Data Availability Statement

The raw data supporting the conclusions of this article will be made available by the authors, without undue reservation.

## Author Contributions

YQ and WG contributed to conception and design of the study. HL and ZP collected the database. YQ performed the data pre-processing, index calculation, and wrote the manuscript. YQ and HL contributed to visualization. YQ and CL analyzed and interpreted the results. All authors reviewed the results and approved the final version of the manuscript.

## Conflict of Interest

The authors declare that the research was conducted in the absence of any commercial or financial relationships that could be construed as a potential conflict of interest.

## Publisher’s Note

All claims expressed in this article are solely those of the authors and do not necessarily represent those of their affiliated organizations, or those of the publisher, the editors and the reviewers. Any product that may be evaluated in this article, or claim that may be made by its manufacturer, is not guaranteed or endorsed by the publisher.
